# Application of K and Zn Influences the Mineral Accumulation More in Hybrid Than Inbred Maize Cultivars

**DOI:** 10.3390/plants10102206

**Published:** 2021-10-17

**Authors:** Hafiz Muhammad Ali Raza, Muhammad Amjad Bashir, Abdur Rehim, Qurat-Ul-Ain Raza, Graeme P. Berlyn, Shafeeq Ur Rahman, Yucong Geng

**Affiliations:** 1 Department of Soil Science, Faculty of Agricultural Sciences and Technology, Bahauddin Zakariya University, Multan 60800, Pakistan; maraza1524@gmail.com (H.M.A.R.); quratulain1111@yahoo.com (Q.-U.-A.R.); 2 College of Agriculture, Bahauddin Zakariya University, Bahadur Sub-Campus Layyah, Multan 60800, Pakistan; amjad.bashir941@gmail.com (M.A.B.); malikshafeeq1559@gmail.com (S.U.R.); 3 Key Laboratory of Nonpoint Source Pollution Control, Ministry of Agriculture, Institute of Agricultural Resources and Regional Planning, Chinese Academy of Agricultural Sciences, Beijing 100081, China; 4 School of Forestry and Environmental Studies, Yale University, New Haven, CT 06511, USA; graeme.berlyn@yale.edu; 5 Farmland Irrigation Research Institute, Chinese Academy of Agricultural Sciences, Xinxiang 453003, China

**Keywords:** membrane stability index, maize genotypes, zinc, nutrient, plant physiology

## Abstract

Maize (*Zea mays* L.) is an important crop used for feeding humans and cattle globally. Deficiency of potassium (K) and zinc (Zn) adversely impacts the maize crop productivity and quality. However, the application of these nutrients shows variant responses in different maize cultivars. To understand this perspective, the current study aimed at investigating K and Zn’s optimal concentration in different hybrid and inbred maize cultivars. The treatments were based on three zinc levels (0, 6, and 12 mg Zn kg^−1^) and K levels (0, 30, and 60 mg kg^−1^), and their respective combinations. The experiment results showed that combined fertilization approaches of Zn and K (Zn12K60) improved the plant biometric, and physiological attributes of maize crop. The results revealed a significant increase in plant height (45%), fresh weight (70%), and dry weight (45%). Similarly, physiological attributes significantly improved the relative water content (76.4%), membrane stability index (77.9%), chlorophyll contents (170%), and photosynthetic rate (130%) in both inbred and hybrid genotypes. Furthermore, Zn and K (Zn12K60) increased transpiration rate (E), stomatal conductance (Ci), and internal CO_2_. In conclusion, maize hybrids (Neelam and DK-6142) were observed best compared with inbred (Afghoi and P-1543) cultivars with the combined application of Zn and K (Zn12K60). Thus, these inbred varieties should be preferred for fodder requirement with optimum fertilizer (Zn12K60) application in Zn deficient soils.

## 1. Introduction

*Zea mays* L. is considered an important crop worldwide for feeding animals and humans. In Pakistan, it is the third major cereal crop after wheat and rice. Maize belongs to the family Poaceae, and is considered a major stable crop due to its high human consumption, livestock feeding, and industrial usage. During 2020–2021, maize production increased approximately 7.4% due to the rise in areas under maize cultivation in Pakistan [1,2]. The consumption of maize is not limited to humans but is also used as bioenergy and fodder for livestock [3]. Important constituents of maize fodder that makes it a good diet for animals includes protein contents, crude fiber contents, ash contents, soluble carbohydrates, and K and Zn contents [4].

Potassium (K) is an important macronutrient after N and P for all crops, especially for cereal crops [5] because it performs a variety of functions in plants such as enzymatic activation, protein synthesis, xylem, phloem transport system, stomatal regulation, and maintaining the balance between cation and anion and showing resistance against different stresses [6]. It poses an influence on the root growth and salt tolerance at the early growth stage of maize and performs a key role in the enhancement of shoot and root biomass [7]. In maize, K deficiency symptoms are not shown clearly, but it may cause serious damage to maize production [8]. Pakistani soils are alkaline to calcareous and contain a reasonable concentration of K. Due to more use of tube well water, intense cropping pattern, complete removal of straw from the field, and K immobilization in clay minerals there is a K deficiency [9,10].

Zinc (Zn) is a crucial micronutrient due to its significant roles in the enzymatic reactions essential for photosynthesis [10]. Moreover, it contributes to enhancing the germination rate, improving the quality of the products, and increased crop productivity per unit area. It helps in maximizing the catalytic activities to promote growth and development at principal growth stages [11]. Zn supplementation can increase plant growth, and yield. It helps in plant disease management, limiting the contaminants uptake, and helps tolerate environmental stress [11,12,13]. Furthermore, Zn uptake also improves due to various soil factors, and improves Zn sorption and desorption, respectively [14]. It is also involved in improving early germination and establishment and plant growth when high Zn concentration is applied [15].

An insufficient amount of Zn severely reduces the yield of maize crop grown in the world’s temperate areas. However, the behavior of different maize hybrids varies in the uptake of available nutrients [12]. In the rice–wheat rotation system, the depressive effect of phosphorous (P) on Zn is reduced by increasing the level of K [13]. The combined application of Zn and K significantly increased the K uptake in maize [2]. The soils with the marginal deficiency of K and Zn can provide more yield of maize crop with the application of K and Zn fertilizers [2,4]. A recent study indicated that co-fertilization of K and Zn influenced maize production effectively [2]; the need of the current study was raised to identify the role of their interaction both in inbred and hybrid cultivars. As maize is susceptible to Zn deficiency, the current research was planned to analyze the potential of hybrid and inbred maize genotypes response against the different rates of Zn and K application on crop growth and physiology in zinc-deficient soils. The objectives of the current study were to: (1) identify the nutrient–nutrient interaction of K and Zn in an agro-system, (2) measure the influences of maize physiological characteristics with combined application, and (3) investigate the behavior of different cultivars under K and Zn combined fertilizer application.

## 2. Materials and Methods

### 2.1. Pot Experiment

#### 2.1.1. Experimental Soil Details

To evaluate the influence of K and Zn fertilizers, a pot experiment was carried out at the research farm of the Department of Soil Science, Faculty of Agricultural Sciences and Technology, Bahauddin Zakariya University, Multan (30.258° N, 71.515° E). The soil used in the experiment was analyzed to determine the physicochemical properties such as electrical conductivity (0.52 dS m^−1^), pH (7.96), organic matter (0.45%), extractable potassium (0.68 ± 0.5 mg kg^−1^), calcium carbonates (4.58%), and AB-DTPA extractable Zn (0.85 mg kg^−1^). The soil used in this experiment was low in plant-available Zn (0.68 mg kg^−1^). However, the soil texture was loam (sand: 43.6%, silt: 38.7%, clay: 19.3%). The soil pH and electrical conductivity was measured with soil extract and saturated paste. Organic matter was analyzed using the Walkley–Black method [14], the extractable potassium by [15], the calcium carbonate by acid dissolution [16], and AB-DTPA extractable Zn using an atomic absorption spectrophotometer [17], respectively.

#### 2.1.2. Experiment Details

Four maize genotypes, P-1543, DK-6142 (hybrid genotypes), Pak Afghoi and Neelam (inbred genotype), each with three replications were used in this experiment. These cultivars are locally common and highly adopted to the climatic conditions. Pots were filled with 8 kg of soil and organized in a completely randomized block design (CRD) with the factorial arrangement. Three Zn levels (0, 6, and 12 mg Zn kg^−1^) and three K levels (0, 30, and 60 mg kg^−1^) were applied. Whereas ZnSO_4_.7H_2_O and K_2_SO_4_ were used as a source of Zn and K. Nitrogen and P were applied in the form of urea and di-ammonium phosphate (DAP).

Seven seeds were sown in each pot. After two weeks of germination, only three plants were maintained in each pot. During the whole experiment period, the moisture level was maintained at field capacity. After fifteen and twenty-five days of germination, the 2nd and 3rd dose of nitrogen was applied. Seedlings were harvested after thirty-five days of germination (inflorescence emergence stage, BBCH-51) as it fulfills the aim of plant physiological study, then washed with distilled water. After sun drying, the samples were oven-dried at 65 ± 5 °C for 48 h to obtain a constant weight. Then well-ground samples were digested by a di-acid mixture (HNO_3_:HCLO_4_ in 2:1 ratio). To determine the Zn concentration from digested samples an atomic absorption spectrophotometer (Thermo Scientific 3000 Series, Waltham, MA, USA) was used, while a flame photometer was used to determine the K concentration.

### 2.2. Plant Analysis

#### 2.2.1. Determination of Morphological Attributes of Maize Crop

Morphological parameters such as plant height (cm), shoot fresh weight (g), and shoot dry weight (g) were measured at principal growth stage 3, stem elongation (BBCH-39) [18], using a measuring tape and weighing balance.

#### 2.2.2. Physiological and Gas Exchange Parameters

Some physiological parameters such as relative water contents (RWC) were measured by taking the difference of fresh weight and turgid weight as provided below.

(1) RWC=(fresh weight−dry weight)/(turgid weight−dry weight) × 100

While the membrane stability index (MSI) was measured by the formula [19] as:

(2) $$MSI=\left\{1-\left(\frac{C1}{C2}\right)\right\}\;\times \;100$$

Chlorophyll contents were measured at principal growth stage 3, stem elongation (BBCH-39), with the help of a chlorophyll meter (SPAD value).

On the other hand, gas exchange parameters such as the photosynthetic rate (A), transpiration rate (E), stomatal conductance (gs), and internal CO_2_ (Ci) were also measured at the principal growth stage 3, stem elongation (BBCH-39), with the help of an infrared gas analyzer (IRGA; Analytical Development Company, Hoddesdon, UK).

#### 2.2.3. Nutrient Concentration

For the determination of nutrient concentration in plant samples, the oven dried samples were ground and digested using a di-acid mixture (HNO_3_:HCLO_4_ in 2:1 ratio). The filtrate was prepared and the ionic contents, such as Zn and K concentrations, in the plant samples were measured with the help of an atomic absorption spectrophotometer (Thermo Scientific 3000 Series, Waltham, MA, USA), and flame photometer (FP 6410, Shanghai Jingke, China), respectively [15].

### 2.3. Statistical Analysis

A completely randomized design (CRD) with the factorial arrangement was the basis of the analysis of variance (ANOVA). The analyzed data were compared by an LSD test at a 5% probability level. A statistical software was used (Statistix 9) on windows for statistical analysis.

## 3. Results

### 3.1. Effect of K and Zinc on Biometric Attributes

A significant increase was observed in all biometric attributes of maize crop through combined application of Zn and K, (Zn12K60) among all cultivars in relation to control conditions (K0Zn0). Plant height was significantly improved with the application of Zn12K60 in inbred Afghoi (118.2 cm), followed by Neelam (112.80 cm).

Plant shoot fresh weight was increased in inbred Afghoi (83.70 g) followed by P-1543 (73.10 g). Ranges for shoot fresh were 47.86–71.7 g pot^−1^ for Neelam and 49.16–83.7 g pot^−1^ for Afghoi, while ranges for hybrid cultivars were 47.56–65.83 g pot^−1^ and 45.5–71.1 g pot^−1^ for DK-6142 and P-1543, respectively. However, shoot dry weight was significantly improved with the application of (Zn12K60) in inbred Afghoi (16.17 g) followed by Neelam (14.07 g). A significant increase was observed for both shoot fresh and dry weight through the combined application of Zn and K (Zn12K60) in comparison with control (Table 1).

### 3.2. Effect of K and Zn on Physiological Attributes

A significant effect on physiological parameters (relative water content, membrane stability index, and chlorophyll contents) of maize crop was observed due to combined application of Zn and K (Zn12K60) among all the cultivars and their interaction compared with the control (Table 2). In the case of inbred cultivars (Neelam and Afghoi), Neelam performed better than Afghoi, while in hybrids (DK-6142 and P-1543) DK-6142 performed better than P-1543. The relative water content (RWC) was maximum for Neelam (89.31%) followed by DK-6142 (88.08%) with combined application of Zn and K (Zn12K60) with respect to the control (Table 2).

Regarding the membrane stability index and chlorophyll contents, inbred and hybrid maize cultivars (Neelam and DK-6142) performed better under combined fertilizer applications than Afghoi and P-1543. Due to the combined application of Zn and K (Zn12K60), maximum values for the membrane stability index were observed in the Neelam cultivar (94.40%), followed by the Neelam cultivar (91.87%) with Zn0K60. Whereas the highest value of chlorophyll contents (SPAD value) was observed for DK-6142 (54.54) followed by P-1543 (49.40), (Table 2).

### 3.3. Effect of K and Zn on Gas Exchange and Nutrient Contents (K and Zn)

Similar to other parameters, gas exchange parameters such as photosynthetic parameter (A), transpiration rate (E), stomatal conductance (gs), and internal CO_2_ concentration (Ci) also play a vital role in plant growth and development. The combined application of K and Zn (K60Zn12) showed a significant effect on the gas exchange (A, E, gs, and Ci) parameter of both inbred and hybrid cultivars of maize crop compared with the control (Table 3 and Table 4). Maximum values for A were observed with Zn12K60 in DK-6142 (38.13 µmol m^−2^ s^−1^), followed by DK-6142 (35.40 µmol m^−2^ s^−1^) with Zn6K60 and P-1543 (34.80 µmol m^−2^ s^−1^) with Zn12K60.

Whereas E was improved in Neelam (12.28 mmol m^−2^ s^−1^) and Afghoi (10.86 mmol m^−2^ s^−1^) with a combined application of K and Zn (Zn12K60). Furthermore, the gs, and Ci were significantly improved in DK-6142 (0.39 mmol m^−2^ s^−1^; 353.5 µmol mol^−1^) and P-1543 (0.36mmol m^−2^ s^−1^; 332.2 µmol mol^−1^), respectively, with the combined application of K and Zn (K60Zn12); more so than under control conditions where no Zn and K were applied (Table 3 and Table 4).

The nutrients including K and Zn concentration in the shoot of maize crop varied among genotypes. The K concentration in inbred (Neelam and Afghoi) cultivars of maize crop ranged from 1.24 to 2.43%, while it ranged from 1.35 to 2.54% for hybrid (DK-6142 and P-1543) genotypes. Shoot K concentration was significantly improved in DK-6142 (2.54 mg kg^−1^) and P-1543 (2.52 mg kg^−1^).

The Zn concentration in shoot samples of inbred maize cultivars, ranged from 17.23 to 29.73 mg kg^−1^, while it ranged from 18.13 to 30.63 mg kg^−1^ for hybrid cultivars. Shoot Zn concentration was significantly increased with Zn12K30 application in DK-6142 (31.4 mg kg^−1^) followed by P-1543 (30.7 mg kg^−1^) for inbred and hybrid genotypes, respectively; more so than under control conditions (Zn0K0; Table 4).

## 4. Discussion

The genomic variations among crop plants provide a valuable tool in selecting the cultivars with desirable characteristics. In the present study, due to a deficiency of Zn in alkaline calcareous soils, the combined application of Zn + K improved the biometric and physiological attributes of all maize cultivars. The increase in biometric characteristics of maize crop was due to the involvement of Zn and K in different physiological processes of the plant such as stomatal regulation, activation of the enzyme, chlorophyll synthesis, cell osmosis, and an increase in water adsorption [5,11,20] that are involved in promoting the plant growth and development.

An increase in biometric attributes of maize crop is due to the combined application of Zn and K, because Zn also performs a vital role in the metabolic process, carbohydrates transformation, and enzymatic activation and protein synthesis [4]. According to [21], K application perhaps caused the greater mobilization of nutrients both in plant and soil, which ultimately involved in increasing the green and dry matter yield by improving the photosynthesis process and enzymatic activities.

The relative water contents remain high at the vegetative stage but low at maturity and play a vital role in the proper growth and functioning of the plant body [22]. Moreover, lower relative water contents cause a decrease in growth due to a reduction in leaf area. Application of K and Zn significantly increased the membrane stability index of wheat cultivars, due to the combined application of K and Zn, which improved the antioxidant enzymatic [23].

The combined application of K and Zn (K60Zn12) has a significant effect on the gas exchange (A, E, gs, and Ci) parameter of both inbred and hybrid cultivars of maize crop than under control conditions. As both the nutrients are involved in the improvement of water relations with chlorophyll contents, they encourage the improvement of many biochemical and physiological processes [24]. Zinc application manipulates more influence on chlorophyll formation and carbonic anhydrase activity, which is involved in assisting the diffusion of CO_2_ towards chloroplast from the liquid phase of a cell [25], which ultimately endorses the photosynthetic rate. The concentration of K and Zn performs an important role in the opening and closing of stomata and controls the process of photosynthesis [23]. The improved physiological characteristics perform a positive part to improve the biometric parameters and yield of maize crop [26]. This can be the reason to improve the photosynthetic rate and to overcome the negative influences due to abiotic stresses. The application of K and Zn can improve the plant biomass, which that might be associated with the higher enzymatic actions and enhanced physio-chemical processes [27].

The application of Zn dramatically increased the seedling growth and dry biomass due to an increase in photosynthesis [2,5]. Zinc application is also involved in improving the transpiration rate, the deficiency reduced the instantaneous transpiration efficiency of a leaf [28]. Moreover, Zn nutrition also performs a vital role in enhancing water use efficiency. About a 20–25% increase was observed due to proper nutrient practices [29]. The activity of Zn is highly involved in the improved production and quality enhancement of the obtained seed [30].

The application of K and Zn increased the K and Zn contents of maize crop. Both of these nutrients have a key role in maize growth, and similar results were reported [31]. The uptake of K by maize crop was not affected significantly due to sources of Zn, but a higher value of K uptake was recorded with the ZnSO_4_ application. In addition, the uptake of K by maize crop was significantly increased with increasing levels of Zn up to 6 kg Zn ha^−1^ [32].

## 5. Conclusions

The current study concludes that combined fertilization of K and Zn produced a significant effect on the biometric, physiological, and gaseous exchange characteristics of four maize genotypes. However, the integrated application of Zn and K (Zn12K60) maize hybrids Neelam and DK-6142 performed best as compared with Afghoi and P-1543. It is recommended that hybrid genotypes should be preferred for under alkaline calcareous conditions, where deficiency of Zn is common, along with the application of Zn and K because they increase the efficiency of each other. Whereas the mechanism and the result under different soil types and climatic conditions is a research gap. Moreover, the grain yield must be studied with such nutrient application.

## Figures and Tables

**Table 1:** Effect of K and Zn on biometric attributes.

Treatment	Cultivar	Genotypes	Plant Height (cm)	Plant Fresh Weight (cm)	Plant Dry Weight (cm)
K0Zn0	Inbred	Neelam	62.00 ± 3.3 u	51.20 ± 2.2 mn	10.63 ± 0.61 o–r
		Afghoi	69.53 ± 2.9 t	55.83 ± 3.4 mn	11.30 ± 0.55 k–o
	Hybrid	DK-6142	59.67 ± 3.4 u	50.23 ± 1.7 mn	10.07 ± 0.46 rs
		P-1543	58.00 ± 3.6 u	49.17 ± 2.3 n	9.90 ± 0.24 s
K0Zn6	Inbred	Neelam	79.37 ± 2.4 p–s	56.17 ± 3.7 jk	11.40 ± 0.52 k–n
		Afghoi	82.97 ± 5.3 n–r	61.53 ± 2.8 e–h	12.30 ± 0.61 f–i
	Hybrid	DK-6142	77.03 ± 4.2 rs	54.27 ± 3.2 j–l	10.90 ± 0.43 n–q
		P-1543	74.70 ± 4.7 st	51.43 ± 1.6 lm	10.23 ± 0.38 q–s
K0Zn12	Inbred	Neelam	86.90 ± 5.5 l–o	57.77 ± 3.4 g–j	11.67 ± 0.44 i–l
		Afghoi	93.17 ± 6.4 i–l	63.80 ± 4.1 d–f	12.43 ± 0.56 d–h
	Hybrid	DK-6142	84.23 ± 3.8 n–q	56.67 ± 2.7 ij	11.33 ± 0.49 k–n
		P-1543	83.23 ± 3.5 n–r	54.53 ± 3.3 j–l	10.93 ± 0.35 m–p
Zn0K30	Inbred	Neelam	81.80 ± 4.3 o–r	56.50 ± 2.9 j	11.50 ± 0.58 j–n
		Afghoi	84.53 ± 6.2 m–p	62.17 ± 4.6 e–g	12.40 ± 0.77 e–h
	Hybrid	DK-6142	79.47 ± 2.7 p–s	54.73 ± 1.3 j–l	11.00 ± 0.38 l–o
		P-1543	77.80 ± 2.5 q–s	51.83 ± 1.7 k–m	10.30 ± 0.29 p–s
Zn6K30	Inbred	Neelam	97.77 ± 4.6 f–i	61.67 ± 2.5 e–h	12.40 ± 0.41 e–h
		Afghoi	102.73 ± 7.7 e–g	69.07 ± 3.2 bc	13.60 ± 0.64 bc
	Hybrid	DK-6142	96.43 ± 5.3 g–j	61.17 ± 3.3 f–i	12.17 ± 0.33 g–j
		P-1543	95.10 ± 3.8 h–k	63.47 ± 3.7 d–f	12.60 ± 0.53 d–g
Zn12K30	Inbred	Neelam	103.47 ± 6.5 d–f	64.63 ± 4.1 c–f	12.77 ± 0.73 d–g
		Afghoi	107.60 ± 2.4 b–e	72.17 ± 4.8 b	13.97 ± 0.47 b
	Hybrid	DK-6142	101.13 ± 5.7 e–h	62.10 ± 3.6 e–g	12.70 ± 0.66 d–g
		P-1543	102.13 ± 4.4 e–g	65.83 ± 4.2 c–e	12.87 ± 0.36 d–f
Zn0K60	Inbred	Neelam	91.03 ± 2.6 j–m	58.50 ± 1.9 g–j	11.87 ± 0.29 h–k
		Afghoi	97.73 ± 5.3 f–i	65.93 ± 2.7 c–e	12.87 ± 0.55 d–f
	Hybrid	DK-6142	89.03 ± 4.8 k–n	57.20 ± 1.7 h–j	11.40 ± 0.32 k–n
		P-1543	87.70 ± 5.2 l–o	58.30 ± 2.3 g–j	11.60 ± 0.28 j–m
Zn6K60	Inbred	Neelam	106.53 ±7.4 b–e	66.07 ± 3.5 c–e	13.03 ± 0.76 c–e
		Afghoi	112.50 ± 8.3 ab	79.50 ± 3.7 a	15.93 ± 0.83 a
	Hybrid	DK-6142	104.53 ± 3.6 c–e	63.13 ± 2.8 d–f	12.50 ± 0.44 d–h
		P-1543	105.53 ± 4.5 c–e	67.10 ± 2.9 cd	13.10 ± 0.59 b
Zn12K60	Inbred	Neelam	112.80 ± 7.7 ab	71.70 ± 3.2 b	14.07 ± 0.65 b
		Afghoi	118.20 ± 6.8 a	83.70 ± 4.4 a	16.13 ± 0.88 a
	Hybrid	DK-6142	109.47 ± 4.7 b–d	65.83 ± 4.1 c–e	12.87 ± 0.33 d–f
		P-1543	110.47 ± 5.6 bc	73.10 ± 2.9 b	14.00 ± 0.74 b

Note: All values are given as means ± standard deviations. The lettering indicates the significance difference with reference to LSD test (5%).

**Table 2:** Effect of K and Zn on physiological attributes.

Treatment	Cultivar	Genotypes	RWC (%)	MSI (%)	Chlorophyll Contents (SPAD Value)
K0Zn0	Inbred	Neelam	38.62 ± 1.8 u	59.17 ± 1.4 r	0.48 ± 0.9 r
		Afghoi	35.96 ± 1.9 u	53.50 ± 1.8 s	17.82 ± 0.8 r
	Hybrid	DK-6142	48.97 ± 1.2 s	52.65 ± 1.3 s	20.17 ± 0.8 r
		P-1543	43.56 ± 3.1 t	50.99 ± 1.0 s	18.93 ± 0.7 r
K0Zn6	Inbred	Neelam	51.09 ± 1.3 rs	69.87 ± 1.5 m–o	26.79 ± 0.9 pq
		Afghoi	48.47 ± 2.7 s	62.33 ± 1.1 qr	25.46 ± 1.2 q
	Hybrid	DK-6142	61.23 ± 2.3 l–n	62.89 ± 1.3 qr	29.04 ± 1.6 n–q
		P-1543	58.28 ± 0.7 n–p	59.55 ± 1.7 r	26.71 ± 1.3 pq
K0Zn12	Inbred	Neelam	55.20 ± 1.8 p–r	74.57 ± 3.4 i–m	30.94 ± 2.1 l–o
		Afghoi	54.36 ± 2.8 p–r	68.23 ± 2.0 n–p	27.94 ± 1.3 o–q
	Hybrid	DK-6142	67.35 ± 0.3 ij	68.38 ± 1.8 n–p	32.08 ± 1.9 k–n
		P-1543	64.15 ± 3.4 j–m	65.05 ± 2.4 o–q	30.08 ± 2.2 l–p
Zn0K30	Inbred	Neelam	52.68 ± 2.0 q–s	70.30 ± 2.3 l–n	27.21 ± 1.6 o–q
		Afghoi	50.06 ± 3.7 s	62.83 ± 1.8 qr	25.54 ± 1.5 q
	Hybrid	DK-6142	62.91 ± 1.0 k–m	63.27 ± 1.9 p–r	29.82 ± 2.2 m–p
		P-1543	59.95 ± 1.8 m–o	59.98 ± 1.6 qr	27.16 ± 1.9 pq
Zn6K30	Inbred	Neelam	68.34 ± 2.9 ij	82.13 ± 3.6 f–h	37.89 ± 2.8 g–i
		Afghoi	66.87 ± 4.2 jk	75.80 ± 3.3 i–k	35.56 ± 1.6 i–k
	Hybrid	DK-6142	76.48 ± 2.3 e–g	78.09 ± 2.6 h–j	40.29 ± 2.1 f–h
		P-1543	73.19 ± 3.5 gh	74.59 ± 2.3 i–m	36.62 ± 1.7 h–j
Zn12K30	Inbred	Neelam	74.29 ± 3.0 f–h	88.10 ± 4.0 b–d	41.37 ± 2.4 fg
		Afghoi	71.25 ± 1.8 hi	82.77 ± 2.7 f–h	38.70 ± 1.9 g–i
	Hybrid	DK-6142	80.80 ± 1.6 cd	81.20 ± 2.5 f–h	46.83 ± 2.5 b–d
		P-1543	77.11 ± 4.1 d–g	78.86 ± 1.4 hi	42.83 ± 2.9 ef
Zn0K60	Inbred	Neelam	60.71 ± 1.3 m–o	79.37 ± 2.8 g–i	32.84 ± 1.4 k–m
		Afghoi	56.86 ± 3.7 o–q	71.70 ± 1.6 j–n	33.84 ± 1.7 j–l
	Hybrid	DK-6142	73.36 ± 1.0 gh	75.07 ± 2.5 i–l	37.94 ± 2.2 g–i
		P-1543	65.27 ± 2.2 j–l	73.07 ± 1.5 j–n	32.64 ± 1.5 k–n
Zn6K60	Inbred	Neelam	80.45 ± 1.4 c–e	91.87 ± 1.9 ab	45.87 ±2.6 b–e
		Afghoi	77.08 ± 4.4 d–g	85.20 ± 2.5 d–f	43.47 ± 2.8 d–f
	Hybrid	DK-6142	83.92 ± 2.1 bc	85.90 ± 2.7 c–f	49.43 ± 1.9 b
		P-1543	77.84 ± 2.7 d–f	77.78 ± 1.5 h–j	42.89 ± 1.6 ef
Zn12K60	Inbred	Neelam	89.31 ± 2.2 a	94.40 ± 2.2 a	48.28 ± 2.7 bc
		Afghoi	83.11 ± 3.2 c	89.73 ± 1.4 a–d	45.61 ± 1.8 c–e
	Hybrid	DK-6142	88.08 ± 3.8 ab	90.70 ± 2.3 a–c	54.54 ± 1.4 a
		P-1543	82.43 ± 3.2 c	84.37 ± 1.9 e–g	49.40 ± 1.8 a

Note: All values are given as means ± standard deviations. The lettering indicates the significance difference with reference to LSD test (5%).

**Table 3:** Effect of K and Zn on gas exchange parameters.

Treatment	Cultivar	Genotypes	Photosynthetic Rate (µmol m^−2^ s^−1^)	Transpiration Rate (mmol m^−2^ s^−1^)	Stomatal Conductance (mmol m^−2^ s^−1^)
K0Zn0	Inbred	Neelam	15.73 ± 1.56 qr	3.85 ± 0.19 p	0.12 ± 0.03 q
		Afghoi	13.73 ± 1.03 r	3.30 ± 0.24 pq	0.08 ± 0.01 r
	Hybrid	DK-6142	19.40 ± 0.95 no	3.19 ± 0.12 pq	0.153 ± 0.02 m–p
		P-1543	16.73 ± 1.07 pq	2.97 ± 0.09 q	0.13 ± 0.05 pq
K0Zn6	Inbred	Neelam	20.23 ± 1.08 m–o	6.36 ± 0.12 j–n	0.170 ± 0.09 k–o
		Afghoi	18.57 ± 0.86 op	5.64 ± 0.33 m–p	0.14 ± 0.04 o–q
	Hybrid	DK-6142	23.23 ± 1.51 j–l	5.53 ± 0.18 n–p	0.19 ± 0.07 j–l
		P-1543	21.57 ± 0.97 k–n	5.07 ± 0.21 p	0.16 ± 0.04 l–p
K0Zn12	Inbred	Neelam	23.47 ± 0.89 jk	7.12 ± 0.18 h–j	0.22 ± 0.08 h–j
		Afghoi	20.80 ± 1.11 l–o	6.43 ± 0.45 j–m	0.183 ± 0.06 k–m
	Hybrid	DK-6142	25.47 ± 0.96 h–j	6.31 ± 0.39 j–n	0.24 ± 0.06 g–i
		P-1543	24.50 ± 0.89 ij	6.02 ± 0.35 k–o	0.213 ± 0.1 ij
Zn0K30	Inbred	Neelam	20.37 ± 1.05 m–o	6.40 ± 0.35 j–m	0.173 ± 0.09 k–o
		Afghoi	18.70 ± 0.78 op	6.05 ± 0.28 k–0	0.150 ± 0.11 n–p
	Hybrid	DK-6142	23.03 ± 0.83 j–l	5.61 ± 0.36 m–p	0.20 ± 0.05 jk
		P-1543	22.03 ± 1.13 k–m	5.25 ± 0.21 op	0.173 ± 0.11 k–o
Zn6K30	Inbred	Neelam	26.93 ± 1.64 g–i	8.35 ± 0.52 e–g	0.26 ± 0.09 fg
		Afghoi	25.27 ± 1.07 h–j	6.85 ± 0.41 i–k	0.216 ± 0.08 ij
	Hybrid	DK-6142	30.27 ± 1.53 ef	7.77 ± 0.28 gh	0.29 ± 0.16 d–f
		P-1543	27.60 ± 1.23 gh	6.96 ± 0.44 h–j	0.256 ± 0.12 g
Zn12K30	Inbred	Neelam	28.83 ± 0.81 fg	9.37 ± 0.51 d	0.29 ± 0.07 d–f
		Afghoi	27.13 ± 1.33 gh	8.29 ± 0.39 e–g	0.25 ± 0.05 gh
	Hybrid	DK-6142	33.83 ± 1.45 b	8.27 ± 0.47 fg	0.32 ± 0.11 c
		P-1543	30.83 ± 1.49 ef	7.31 ± 0.22 hi	0.30 ± 0.13 c–e
Zn0K60	Inbred	Neelam	23.80 ± 0.95 jk	7.31 ± 0.31 hi	0.22 ± 0.04 h–j
		Afghoi	21.77 ± 0.77 k–n	6.79 ± 0.15 i–l	0.180 ± 0.11 k–n
	Hybrid	DK-6142	27.13 ± 1.17 gh	6.68 ± 0.25 i–l	0.25 ± 0.09 gh
		P-1543	25.47 ± 1.35 h–j	5.97 ± 0.14 l–o	0.215 ± 0.06 ij
Zn6K60	Inbred	Neelam	30.73 ± 1.63 ef	10.49 ± 0.66 bc	0.32 ± 0.11 c
		Afghoi	29.07 ± 1.88 fg	9.52 ± 0.49 d	0.27 ± 0.18 e–f
	Hybrid	DK-6142	35.40 ± 1.19 b	9.14 ± 0.29 de	0.36 ± 0.15 b
		P-1543	32.07 ± 1.58 c–e	8.32 ± 0.35 e–g	0.31 ± 0.06 cd
Zn12K60	Inbred	Neelam	33.47 ± 1.45 b–d	12.28 ± 0.69 a	0.35 ± 0.15 b
		Afghoi	31.13 ± 0.98 d–f	10.86 ± 0.55 b	0.30 ± 0.09 c–e
	Hybrid	DK-6142	38.13 ± 1.12 a	9.85 ± 0.37 cd	0.39 ± 0.14 a
		P-1543	34.80 ± 1.05 b	9.10 ± 0.63 d–f	0.36 ± 0.17 ab

Note: All values are given as means ± standard deviations. The lettering indicates the significance difference with reference to LSD test (5%).

**Table 4:** Effect of K and Zn on nutrient contents.

Treatment	Cultivar	Genotypes	Internal CO_2_ Concentration (µmol mol^−1^)	Shoot Zn (mg kg^−1^)	Shoot K (g kg^−1^)
K0Zn0	Inbred	Neelam	131.8 ± 16 st	18.3 ± 0.7 st	1.28 ± 0.03 op
		Afghoi	110.5 ± 11 t	17.2 ± 1.6 t	1.243 ± 0.01 p
	Hybrid	DK-6142	149.8 ± 13 p–s	19.1 ± 2.4 r–t	1.383± 0.02 lm
		P-1543	135.9 ± 7 rs	18.1 ± 1.6 st	1.35 ± 0.03 mn
K0Zn6	Inbred	Neelam	158.1 ± 20 p–r	22.4 ± 1.5 n–p	1.3 ± 0.04 o
		Afghoi	136.5 ± 17 rs	21.4 ± 2 o–q	1.27 ± 0.01 op
	Hybrid	DK-6142	168.2 ± 04 n–p	23.7 ± 1.3 l–n	1.40 ± 0.06 l
		P-1543	154.3 ± 09 p–s	22.9 ± 0.7 no	1.37 ± 0.03 lm
K0Zn12	Inbred	Neelam	188.4 ± 11 m–o	26.6 ± 3.5 g–j	1.31 ± 0.02 no
		Afghoi	166.4 ± 08 o–q	25.1 ± 2.7 i–m	1.29 ± 0.01 o
	Hybrid	DK-6142	191.4 ± 14 mn	28.6 ± 3.3 c–g	1.41 ± 0.05 l
		P-1543	171.7 ± 12 n–p	27.8 ± 4.4 e–h	1.38 ± 0.04 lm
Zn0K30	Inbred	Neelam	165.7 ± 08 o–q	19.6 ± 0.9 q–s	2.23 ± 0.07 ij
		Afghoi	142.4 ± 11 q–s	18.3 ± 1.8 rs	2.18 ± 0.02 k
	Hybrid	DK-6142	173.1 ± 09 n–p	20.8 ± 2.5 p–r	2.35 ± 0.04 gh
		P-1543	161.5 ± 12 pq	19.8 ± 2.7 q–s	2.35 ± 0.08 gh
Zn6K30	Inbred	Neelam	267.9 ± 13 g–i	24.1 ± 3.7 k–n	2.24 ± 0.02 ij
		Afghoi	245.4 ± 19 i–k	23.1 ± 1.6 m–o	2.21 ± 0.06 jk
	Hybrid	DK-6142	266.4 ± 09 g–j	26.1 ± 4.6 h–k	2.36 ± 0.03 gh
		P-1543	245.5 ±16 i–k	25.3 ± 2.2 i–l	2.33 ± 0.06 h
Zn12K30	Inbred	Neelam	289.2 ± 14 d–g	29.0 ± 2.5 b–e	2.26 ± 0.08 i
		Afghoi	259.2 ± 16 h–j	28.3 ± 5.4 d–g	2.23 ± 0.04 ij
	Hybrid	DK-6142	293.9 ± 19 d–f	31.4 ± 3.3 a	2.37 ± 0.05 d–g
		P-1543	277.2 ± 11 f–h	30.7 ± 1.7 ab	2.34 ± 0.01 gh
Zn0K60	Inbred	Neelam	242.1 ± 09 jk	19.7 ± 2.2 q–s	2.40 ± 0.06 c–f
		Afghoi	221.8 ± 11 kl	18.4 ± 0.8 rs	2.36 ± 0.04 f–h
	Hybrid	DK-6142	224.3 ± 17 kl	21.5 ± 1.4 o–q	2.51 ± 0.02 ab
		P-1543	199.8 ± 07 lm	20.8 ± 3.5 p–r	2.49 ± 0.03 b
Zn6K60	Inbred	Neelam	307.5 ± 20 b–d	25.0 ± 4.6 j–m	2.41 ± 0.07 c–e
		Afghoi	280.0 ± 17 e–h	23.8 ± 5.7 l–n	2.37 ± 0.1 e–h
	Hybrid	DK-6142	329.0 ± 19 a–c	28.5 ± 4.3 d–g	2.52 ± 0.03 ab
		P-1543	304.1 ± 15 c–e	27.2 ± 1.2 f–i	2.50 ± 0.05 b
Zn12K60	Inbred	Neelam	326.2 ± 18 bc	29.7 ± 6.3 a–e	2.43 ± 0.01 c
		Afghoi	304.5 ± 16 c–e	28.1 ± 3.6 d–g	2.41 ± 0.1 cd
	Hybrid	DK-6142	353.5 ± 23 a	30.6 ± 2.4 a–c	2.54 ± 0.06 a
		P-1543	332.2 ± 17 ab	30.0 ± 5.5 a–d	2.52 ± 0.04 ab

Note: All values are given as means ± standard deviations. The lettering indicates the significance difference with reference to LSD test (5%).
